# PRALIMAP: study protocol for a high school-based, factorial cluster randomised interventional trial of three overweight and obesity prevention strategies

**DOI:** 10.1186/1745-6215-11-119

**Published:** 2010-12-06

**Authors:** Serge Briançon, Emilie Bonsergent, Nelly Agrinier, Sabrina Tessier, Karine Legrand, Edith Lecomte, Evelyne Aptel, Serge Hercberg, Jean-François Collin

**Affiliations:** 1 Nancy-University, Paul Verlaine Metz University, Paris Descartes University, EA4360 Apemac, Nancy, France; 2 Nancy-University, Faculty of medicine, School of Public Health, Nancy, France; 3 Nancy University Hospital, Epidemiology and clinical evaluation department, Nancy, France; 4 UMR U557 INSERM/U1125 Inra/CNAM/Paris 13, SBMH-Paris 13 University, Bobigny, France; 5 National conservatory of arts and crafts (CNAM), Nancy, France; 6 Local school office of the Nancy-Metz academy, Nancy, France; 7 Regional institute for health education (IREPS), Nancy, France

## Abstract

**Background:**

Given the increase in overweight and obesity prevalence in adolescents in the last decade, effective prevention strategies for these conditions in adolescents are urgently needed. The PRALIMAP (Promotion de l'ALImentation et de l'Activité Physique) trial aims to evaluate the effectiveness for these conditions of 3 health promotion strategies -- educational, screening and environmental -- applied singly or in combination in high schools over a 2-year intervention period.

**Methods:**

PRALIMAP is a stratified 2 × 2 × 2 factorial cluster randomised controlled trial including 24 state high schools in Lorraine, northeastern France, in 2 waves: 8 schools in 2006 (wave 1) and 16 in 2007 (wave 2). Students entering the selected high schools in the 4 academic years from 2006 to 2009 are eligible for data collection. Interventional strategies are organized over 2 academic years. The follow-up consists of 3 visits: at the entry of grade 10 (T0), grade 11 (T1) and grade 12 (T2). At T0, 5,458 (85.7%) adolescents participated. The educational strategy consists of nutritional lessons, working groups and a final party. The screening strategy consists in detecting overweight/obesity and eating disorders in adolescents and proposing, if necessary, an adapted care management program of 7 group educational sessions. The environmental strategy consists in improving dietary and physical activity offerings in high schools and facilities, especially catering. The main outcomes are body size evolution over time, nutritional behaviour and knowledge, health and quality of life. An evaluation process documents how each intervention strategy is implemented in the schools and estimates the dose of the intervention, allowing for a per protocol analysis after the main intention-to-treat analysis.

**Discussion:**

PRALIMAP aims at improving the prevention and management of overweight and obesity in adolescents by translating current evidence into public health practice. Particular attention is paid to clustering, multiple factorials and long-term duration to address common pitfalls in health promotion trials. The results should inform how best to implement, in a school environment, effective nutrition prevention programs targeting adolescents who are at a point their lives when they develop responsibilities and empowerment for health attitude behaviours.

**Trial registration:**

This trial is registered at ClinicalTrials.gov under NCT00814554.

## Background

Child and adolescence overweight and obesity prevalence has been increasing worldwide during the last decades. Overweight and obesity are considered the most widespread disorders in Europe, affecting, in 2002, approximately 1 in 6 non-adults and in some parts of Europe up to 1 in 3. Adolescents with a body mass index (BMI) equal to or greater than the 85th percentile are at increased risk of obesity in adulthood [1]. Thus, overweight and obesity prevention is an international public health priority requiring the implementation of effective interventions to produce changes in dietary and physical activity patterns in individuals. Two systematic reviews with inconsistent results have been published in this field [2,3], and a recent commentary review explained the discrepant results [4] as being the heterogeneity of the studies in terms of target population, theoretical underpinning, study design and outcome measures.

Only one study in each review targeted adolescents, which confirmed that most programs and studies involve children. However, during adolescence, children are becoming independent and self-determined enough to establish eating habits and physical activity patterns. Besides communities and families, schools have been identified as key settings for public health strategies to lower or prevent the prevalence of overweight and obesity [5]. Fifteen-year-old adolescents spend more time at school than at any other setting outside of the home. The school food offerings potentially have a large impact on adolescents' eating habits because many students, especially those who board full-time or half-time, consume a substantial proportion of their total daily intake at school [6].

Many theoretical considerations underpin the choices, orientations, ways and means of implemented intervention strategies such as healthy eating, nutritional education, physical activity and environmental modifications. Stand-alone interventions or integrated interventions have discrepant effectiveness. The Ottawa charter provides a framework for health promotion actions around 5 means, of which 3 are particularly relevant in this field and context: develop personal skills, reorient health services and create supportive environments [7]. The contribution of each to overweight and obesity prevention alone and in combination has not been extensively explored. Such information would be of great interest for improving public health policies. In 2001 in France, the government set up a National Nutrition and Health Program ("Programme National Nutrition Santé", PNNS) to enhance the global health status of the population by improving nutrition. One of the main objectives was a 20% reduction in excess weight and obesity prevalence among adults and to stop the increase in obesity prevalence among children and adolescents [8]. Research results are awaited the plan renewal.

A powerful trial with an appropriate design - namely clustering and factorization -- and with wide outcomes from knowledge to anthropometric measurements is needed to measure the long-term impact of such health promotion strategies among adolescents in schools. The present report describes the design, implementation and baseline characteristics of clusters and participants of the PRALIMAP (Promotion de l'ALImentation et de l'Activité Physique) trial, a 2 × 2 × 2 factorial cluster, school-based randomised intervention trial testing the effectiveness of 3 overweight and obesity prevention strategies in adolescents.

### Objectives

The main objective of the PRALIMAP trial is to evaluate the effectiveness of 3 public health interventional strategies -- educational, screening, environmental -- applied alone or in combination over a 2-year intervention period to promote healthy dietary and physical activity for adolescents in high school. Adolescent-centred outcomes include nutritional knowledge, attitudes and behaviours; body size; and health-related quality of life (HRQoL).

The secondary objective is to evaluate the process and especially the feasibility of each strategy applied in the high school setting.

## Methods

### Design of the PRALIMAP trial

PRALIMAP is a stratified 2 × 2 × 2 factorial cluster randomised controlled trial. The units of randomisation are state high schools; 24 high schools participated in the trial in 2 waves: 8 in 2006 (wave 1) and 16 in 2007 (wave 2). The interventional strategies are organized by 2 academic years, and follow-up consists of 3 visits.

The PRALIMAP trial has been approved by the French consultative committee for treatment of information in health research (n°06.376) and the French data protection authority (n°906312). This trial is registered at ClinicalTrials.gov under NCT00814554 http://clinicaltrials.gov/ct2/show/NCT00814554.

### Study setting and high school recruitment

In 2006, the Lorraine region, northeastern France, included 4 administrative departments, two of which being mainly rural area (Meuse and Vosges). It counted 2,34 billions inhabitants among whom 154,365 were adolescents aged of 14-18 years old with a higher proportion of boys (51%, n = 79,246). Among these adolescents, 57% (n = 88,076) were attending 203 high schools of which 124 were state schools (n = 80,935 students) and 79 were independent schools (n = 7,141 students). Of the state high schools, 46 were general and technological high schools, with 57,943 students: 14 were in Meurthe-et-Moselle, 1 in Meuse, 22 in Moselle and 9 in Vosges. The remaining 78 state high schools were oriented toward vocational secondary education (i.e., providing practice-oriented education for a specific occupation), with 22,992 students: 22 in Meurthe-et-Moselle, 7 in Meuse, 34 in Moselle and 15 in Vosges.

In 2007, 79,376 students were attending 122 state high schools in the 4 departments. Of the state high schools, 60 were general and technological high schools, with 57,284 students: 17 in Meurthe-et-Moselle, 4 in Meuse, 28 in Moselle and 11 in Vosges. The remaining 62 state high schools were vocational high schools, with 22,092 students: 18 in Meurthe-et-Moselle, 4 in Meuse, 27 in Moselle and 13 in Vosges.

The only eligibility criteria for high school were to be a state administrative establishment (n = 124). The PRALIMAP trial group randomly selected 24 after stratification on department and type of education (general and technological or vocational) for participation in the PRALIMAP trial:

- 5 general and technological and 3 vocational high schools in Meurthe-et-Moselle

- 5 general and technological and 3 vocational high schools in Moselle

- 3 general and technological high schools and 1 vocational high school in Meuse

- 3 general and technological high schools and 1 vocational high school in Vosges

Every selected high school headmaster accepted to participate.

The stratification warranted a well-balanced representativeness on the two used criteria which are known to be associated to body size and nutritional knowledge, attitudes and behaviours.

### Randomisation and student recruitment

**Figure 1:**
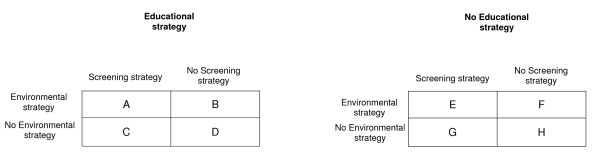
**Randomisation in the PRALIMAP trial with a factorial plan 2 × 2 × 2**. A, B, C, D, E, F, G and H: randomisation groups in the PRALIMAP trial.

The 24 high schools were assigned to receive the 3 strategies according to a 2 × 2 × 2 factorial cluster (high school) randomisation as described in Figure 1. Stratification was on department and type of education. In total, 8 groups, with 3 high schools in each group, were assigned to receive the following interventions:

The 3 strategies (group A)

Educational and environmental strategies (group B)

Educational and screening strategies (group C)

Screening and environmental strategies (group E)

Educational strategy alone (group D)

Environment strategy alone (group F)

Screening strategy alone (group G)

No intervention (group H)

All students of the participating high schools who were registered in the grades targeted by the PRALIMAP trial were likely to be enrolled (Table 1).

**Table 1:** Number of new students entering the selected schools each year in the grade of interest

		*Academic year*				
		2006	2007	2008	2009	Total
***Grade of interest***	**Grade 10**	2,343	*4,028*			**6,371**
	
	**Grade 11**		312	*547*		**859**
	
	**Grade 12**			207	*331*	**538**

	**Total**	**2,343**	**4,340**	**754**	**331**	**7,768**

Wave 1 data are underlined, *wave 2 *data are italicized

Note: New students entering the selected high schools in the grade of interest for each of the 4 academic years beginning 2006 to 2009 are eligible for the data collection.

### Study Interventions (Table 2)

**Table 2:** Elements of the standard operating procedures for each of the 3 prevention strategies

	First high school year (grade 10)	Second high school year (grade 11)
**Educational strategy**	• 5 hours of lectures on nutritional needs • 2 hours and personal work for groups on nutritional rhythms or environment • Organization of a 1-day or half-a-day PRALIMAP party	• 6 hours of lectures on nutritional environment • 2 hours and personal work for collective groups on influence of medias, eco-citizenship, nutritional security measures and prices of food and drink and physical activity • Organization of a 1-day or half-a-day PRALIMAP party

**Screening strategy**	• 2 simultaneous measurements of height, weight and waist circumference by nurses and completing of self-administered questionnaire by student • Calculation of body mass index (BMI) and of EAT-40[17] and HAD [21] scores • Positive screening = overweight or obesity and high waist circumference • Notification of students with positive screening by nurses and medical professional to explain results • Proposition to participate to external healthcare network	• 2 simultaneous measurements of height, weight and waist circumference by nurses and completing of self-administered questionnaire by student • Calculation of body mass index (BMI) and of EAT-40[17] and HAD [21] scores • Positive screening = overweight or obesity and high waist circumference • Notification of students with positive screening by nurses and medical professional to explain results • Proposition to participate to external healthcare network
	
	• Care management = 7 group educational sessions during 1.5 hours supervised by external healthcare network specialized for nutrition:	
	➢ A first session to inform and answer questions about nutrition and weight supervised by a physician and a dietician	
	➢ Two sessions on food practices supervised by a dietician and a psychologist	
	➢ Two sessions on physical activities practices supervised by a sports educator and a psychologist	
	➢ Two sessions on nutritional changes led by a dietician and supervised by all professionals	

Environmental strategy	• Inventory of sports and collective catering features and facilities as well as available activities through an environmental survey	
	• Improvement of environmental characteristics adhering to the PNNS [8] guidelines standing•	
	• Implementation of new features and activities to improve nutritional environment•	
	• Organization of a 1-day or half-a-day PRALIMAP party	

Three prevention strategies are used. By "Educational strategy", we mean developing personal skills to adopt healthy behaviours in the field of nutrition (diet and physical activity) according to current guidelines [7,8]. By "Screening strategy", we mean measuring, detecting overweight/obesity and eating disorders, and proposing if necessary an adapted care management. By "Environmental strategy", we mean developing favourable and supportive environments for healthy behaviours targeting the catering supply of the school and the school policy.

The 3 strategies are implemented in high schools according to standard operating procedures. All activities are performed over the first 2 high school years (corresponding to grades 10 and 11 in the US educational system) between January and June. These strategies target individual nutritional behaviour by acting directly on student skills (educational strategy and screening strategy) or by changing the school environment (environmental strategy).

The educational and environmental strategies are managed by trained health education professionals external to the high schools, called PRALIMAP monitors, specifically recruited for the trial. The monitors clarify objectives to be reached, propose and initiate activities and accompany and support high school professionals. The screening strategy is managed by public health professionals of Nancy-University, high school nurses and practitioners and an external nutrition health network.

#### Educational strategy

This strategy includes 3 types of activities:

1. Nutrition and physical activity lectures, officially registered in the high school course offerings, are provided by high school teachers of Life Sciences and/or Physical Education. Teachers of other disciplines (e.g., librarian, communication, history and geography teachers) can be added according to school resources. The lectures represent 5 hours during the first high school year and 6 hours for the second high school year distributed according to availability of teachers.

2. Students perform collaborative work with partial supervision by teachers and a PRALIMAP monitor. Students are allowed to discover, exchange and find their own answers to a nutritional rhythm and environment and the influence of environmental pressure on nutritional individual choices (e.g., influence of the media, eco-citizenship, cost) during 2 hours during the first and second high school year.

3. A 1-day or half-a-day PRALIMAP party is organized during the last trimester of every school year to reinforce the learned knowledge about healthy food choices and to be physically active in an atmosphere of conviviality, pleasure and friendship. Several activities are organized (e.g., fun physical activities, games, tests, conferences, food and drink tasting), and the production of collaborative works previously described are appreciated according to the availability of high school staff. All high school professionals and all students are invited to participate in the event.

#### Screening strategy

Weight, height and waist circumference of students are measured twice in a single session by high school nurses in the nurse's office, and the Eating Attitudes Test 40 (EAT-40) and Hospital Anxiety and Depression (HAD) questionnaires are completed. All these data are part of the follow-up visit data collection.

The body weight of students wearing underwear is measured with an accuracy of 0.05 kg by use of a calibrated electronic scale (SECA^®^: model number 873 1321009). The body height of students not wearing shoes is measured by a stadiometer (SECA^®^: reference SECA 214 SEC 01) to the nearest 0.1 cm. The body mass index (BMI) is calculated as weight/height^2 ^from the mean of the above 2 measurements. We used the International Obesity Taskforce (IOTF) age- and sex-specific cut-off values for BMI for thinness grades 1, 2 and 3 [9], overweight and obesity [10], with dataset-specific centiles linked to adult cut-off values. Waist circumference is measured with use of a non-elastic flexible tape (SECA^®^: reference 200 SEC 01) at the level of the bellybutton to the nearest 0.1 cm with the subject in a standing position. We use the McCarthy [11] age- and sex-specific cut-off values to define high waist circumference using dataset-specific centiles linked to adult cut-off values. A positive screening is defined by an overweight or obesity according to BMI and high waist circumference.

An EAT-40 score ≥ 30 (on a 0-120 scale) is used as a cut-off value to identify students suspected of having eating disorders. HAD scores ≥ 11 (on a 0-21 scale) is used as a cut-off value to identify students suspected of having anxiety and/or depression.

Nurses notify students with positive screening orally and in writing, explain the consequences of overweight and the importance of adapted care management, and give them letters containing the screening results, including EAT-40 and HAD scores, one for their parents and one for their general practitioner. As recommended by Nihiser et al. [12], the letter to parents typically includes the child's BMI-for-age percentile, an explanation of the results and recommendations for care management. The adapted care management consists of 7 group educational sessions, offered for 1.5 hours by physicians, dieticians, sport educators and psychologists. These sessions are implemented outside of the high school by an external healthcare network specialized in nutrition or inside the high school by a mobile team if a network is not available in the surrounding territory of the high school. These sessions are funded by the regional health insurance system.

#### Environmental strategy

This strategy aims at extending the range of students' nutritional choices and consists in increasing the availability of fruits, vegetables, bread and dairy products, water and physical activity.

First, an environmental survey compiles an inventory of activities and facilities for sports and catering features and facilities at the high school. Second, the environmental characteristics fitting the PNNS guidelines is improved through activities such as presenting a menu with the food group colours and signs for water distribution, and producing information support on available physical activities. Third, a project committee consisting of high school professionals, including the school headmaster, and the PRALIMAP monitor is in charge of implementing new features and activities to improve the nutritional environment. New projects and features can be funded by the regional council of the academic program. The final activity of the environmental strategy is the PRALIMAP party as described for the educational strategy except that students do not participate to the organization and have no collaborative works to present.

### Outcomes and process data

Outcomes and process data are collected. The outcomes include anthropometric data, nutritional attitudes and behaviours, and perceived health and quality of life. The main endpoint of the PRALIMAP trial is the evolution of overweight and obesity prevalence over the 2 intervention years.

Process data include quantitative and qualitative measures of participation and implementation of the 3 strategies according to all the stakeholders.

#### Outcomes

The outcomes data sources are the Board of Education database, self-administered questionnaires, and anthropometric measures.

##### Data collection

**Figure 2:**
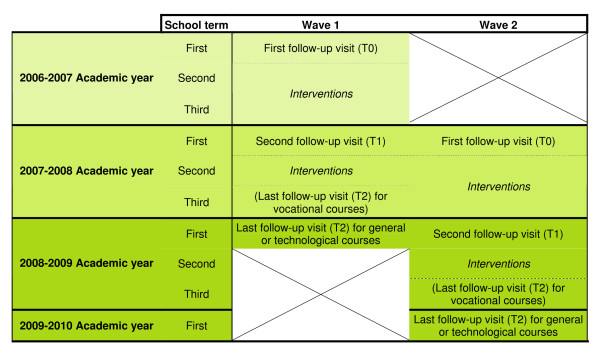
**PRALIMAP trial schedule**.

The 3 follow-up visits (T0, T1, and T2) are carried out at the beginning of the 3 academic years (grades 10, 11 and 12) for general and technological high schools and at the beginning of the 2 academic years and at the end of the second academic year for vocational high schools (Figure 2).

Every academic year, an information letter is sent to the student's parents. Parents must sign a written refusal to collect data for their children. In the high school, students are also given written and oral information. The PRALIMAP monitor explains the purpose of the measurements, reassures students about the confidential nature of data, answers any queries and confirms the right not to participate.

Students entering the selected high schools in the grade of interest in each of the 4 academic years from 2006 to 2009 are eligible for data collection (Figure 2 and Table 1). Students not fluent in reading or writing French or with delayed entry in the high school grade are ineligible.

At each follow-up visit, data on sociodemographic characteristics, nutritional attitudes and behaviours, and perceived health and quality of life are collected by self-administered questionnaires completed in the classroom and merged in a unique report form; body size is measured by trained nurses in the high school nurse's office. Nonattending students are contacted once or twice as necessary for data collection.

##### Sociodemographic characteristics

Sociodemographic characteristics are compiled from the Board of Education database and completed self-administered questionnaires. Data are collected on date of birth, gender, grade, social and professional class of the family head at entry of the student into grade 10 (in 5 groups according to the definition of the French national institute of statistic and economical studies [INSEE]), school boarding status (non-boarder, half-boarder or full boarder), residence (type of residential area, house type, number of people in the home), parents' occupations, adolescent's perception of their parents' weight status and physical activity practice, and family income.

##### Anthropometry

This process involves measuring students' body size according to weight, height, and waist circumference during the follow-up visits and calculating BMI. The international BMI cut-off values [10] are used. The 97th percentile of the Rolland-Cachera curves are also considered [13]. High waist circumference is defined according to McCarthy [11] and/or Katzmarzyk and Lean [14,15]. Overweight and obesity are defined according to BMI cut-off values alone or in combination with waist circumference values. The operating procedures are detailed in the "Screening strategy" paragraph.

##### Nutritional knowledge, attitudes and behaviours

Nutritional knowledge level is obtained by a quiz on dietary guidelines, physical activity, and health and nutrition relationship, for a score ranging from 0 to 100. Attitudes and behaviours are measured with the specifically designed Boire Manger Bouger (BMB; "Drinking, Eating, Moving") questionnaire. Satisfaction with food and physical activity and ability to follow guidelines for fruits and vegetables, dairy products, starchy food, drinks, sugary foods, number of meals and physical activity are explored. The environmental conditions of meals are also investigated.

Physical activity is measured by the International Physical Activity Questionnaire (IPAQ) [16]. The IPAQ assesses the frequency (days per week) and duration (minutes) of sitting and walking and of moderate and vigorous physical activity during the previous 7 days. Physical activity level is thus defined as low, moderate or high (the high level corresponds to nutritional guidelines).

##### Health

The EAT-40 [17], a validated and widely used questionnaire, screens for anorexic and bulimia symptoms. It is a self-reporting questionnaire with responses on a 6-point Likert scale ranging from 0, never, to 6, always. Four dimensions are explored: dieting, bulimia/food preoccupation, oral control and overall eating disorder [18]. Scores are estimated and the cut-off values used are those recommended by the authors.

The HAD [19,20] screens for depression and anxiety with 14 items on a 4-point Likert scale (range 0-3). The psychometric properties in the general population are acceptable [21]. The total score is the sum of the scores on the 14 items, and for each of the 2 subscales, the score is the sum of the scores on the respective 7 items.

The Duke Health profile [22,23], a 17-item generic self-reporting questionnaire explores perceived health and HRQoL with 10 dimensions; the physical, mental, and social dimensions are commonly used. High scores on the 0-100 scale indicate good HRQoL.

To facilitate interpretation, all scores are normalized to a 0-100 scale.

#### Process

Extensive process evaluation is considered a main part of the trial design. This evaluation aims to document how schools assigned to an interventional strategy implement it, and if control schools for this strategy implement interventions related to the theme of this strategy (e.g., environmental interventions implemented in a school that is a control for the environmental strategy). Other main aims are to collect information on the provision and receipt of the 3 nutritional interventions, determine the extent of possible contamination between schools, and report on the experience and impact of the PRALIMAP trial. Thus 2 domains -- implementation and participation -- are explored according to quality and quantity and from 4 points of view: students, PRALIMAP monitors, and school professionals as receivers of information from the PRALIMAP team and as providers of the intervention to students.

The process data sources are observation, stakeholders' interviews, and adolescent self-administered questionnaires.

##### Observation

Members of the research team observe the key processes in the implementation of interventional strategies in every high school and document the processes in activity reports. This observation includes regular meetings with high school professionals and teachers and an annual environmental survey. Meetings are organized once a month, are conducted by the PRALIMAP monitor, and aim to accompany and follow the performance of activities and to uphold the dynamics of the school's investment in the process. As described for the environmental strategy, the PRALIMAP monitor carries out an environmental survey of the headmaster, the financial administrator and the physical education teachers, whatever the strategy assigned to the school, at the beginning of every academic year.

##### Stakeholders' interview

A collective interview (focus group) is carried out with staff responsible for interventional strategies (high school professionals, head teachers) at the end of the 2 intervention years. It is lead by the process experts and psychologists of the PRALIMAP research team. Every PRALIMAP monitor is independently interviewed by use of a semi-structured interview guide by the PRALIMAP process evaluation lead at this time. The aim is to gather information about the content, delivery and stakeholders' appreciation of the intervention strategies over the 2 years (i.e., what was done, what stakeholders liked and disliked, the pros and cons of the interventions, their degree of satisfaction with the program, their appraisal of the benefit for students and recommendations for their own school and others). For the focus group, a full narrative description includes who was present, what was said, interactions between participants, the atmosphere, and the occurrence of significant events such as participants entering or leaving.

##### Student appreciation

A year-specific appreciation questionnaire is included at the T1 and T2 student report form. The survey aims to gain insight into students' perception and evaluation of the PRALIMAP trial (i.e., the school nutritional offerings, interactions with health and high school professionals, PRALIMAP activities participation, what they liked and disliked, how they perceived and incorporated interventional strategies and PRALIMAP as a whole).

### Data management and quality control

A Microsoft Access-based information system was developed to warehouse data (Microsoft Access 2003 v11.5614.6568, Seattle, WA, USA). At baseline, 15 keyboarders in 2006 and 18 in 2007 entered 18,105 and 28,836 data elements, respectively. The mean error rate was 30 per 10,000 data elements.

### Sample size

A total of 6,500 students were expected to attend grade 10 in the 24 high schools participating in the PRALIMAP trial. We anticipated approximately 5,590 participants on the basis of an approximately 86% mean participation rate of students in other nutritional studies [24-26]. Finally, from a sample size of 5,475, an average cluster size of 228 students and an anticipated intra-class correlation coefficient (ICC) of 0.005, we estimated a difference of approximately 4% in prevalence of overweight/obesity between the intervention and non-intervention arms at the end of PRALIMAP trial, assuming an alpha risk of 5% and a power of 80%. Power is assumed to be higher for other endpoints, namely, nutritional knowledge, attitudes and behaviours.

### Planned Analysis

The main judgment criteria consist of body size indicators: overall evolution of overweight and obesity prevalence, and among students with normal body size at trial entry, mean BMI evolution, proportion of students whose BMI evolution curve from baseline to the end of follow-up deviated from the IOTF and French norms for BMI between 16 and 18 years of age. Secondary judgment criteria refer to nutritional knowledge attitudes and behaviours and perceived health and quality of life, namely, the evolution in proportion of adolescents following nutritional guidelines and in mean nutritional knowledge score, the proportion of adolescents with eating disorders and high anxiety or depression scores, and finally Duke physical, mental and social dimensions scores.

Basic descriptive statistics were used to characterize the baseline participant population and interventions at both the participant and cluster levels. To produce accurate estimates of the used indicators in the Lorraine general population attending high schools, students' data were weighted by the product inverse of their high school probability to be included and their probability to participate. Intra-cluster similarity was analyzed by the ICC.

Students leaving high school, as well as students participating in the PRALIMAP over the intervention period will be described by a flow chart according to the CONSORT statement adapted to cluster randomised trials [27,28] and analyzed for possible selection bias.

Both cross-sectional and longitudinal analyses with cluster-specific methods are planned. General Estimating Equations (GEE) models will be used to take in account the hierarchical and longitudinal nature of the data. All analyses are planned at the individual student level on an intention-to-treat basis. Given the complexity of the analysis, details will be described more completely in the future.

The dose of intervention students receive will be estimated by the evaluation process in terms of a score developed by experts and will be taken into account on a per protocol analysis. Details of this analysis will be presented elsewhere.

SAS can accommodate the factorial clustered design and will be used for analysis (SASTM v9.2, SAS Inst., Cary, NC, USA).

### Inclusion data

**Figure 3:**
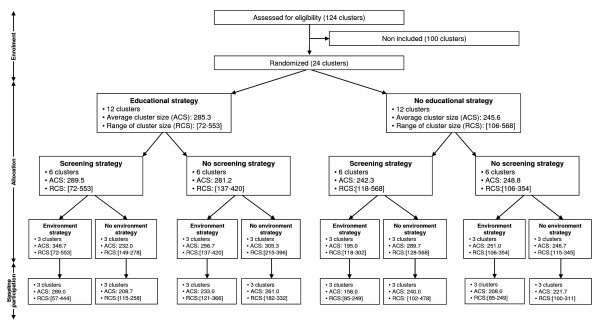
**Flow diagram of the initial phases of the progress of clusters and individuals in PRALIMAP**.

The flow diagram (Figure 3) presents the processing of clusters and students through the initial phases of the PRAMILAP.

At cluster enrolment, the mean overall high school size was 812 students (range 283-1,893 students), and 29% had more than 1,000 students. The mean grade 10 size was 265.5 students (72-568). The mean grade 10 participants cluster size was 227.4 students (57-478). Thus, among the 6,371 grade 10 students, 5,458 (85.7%) underwent at least one baseline measurement, without any difference in participation in anthropometric and self-administered measurements. High school participation rates highly differed (from 72.0% to 99.1%) and were higher in general and technological than vocational high schools (86.6% vs 80.9%, p = < 0.0001) and in the rural administrative department of Vosges than in the other 3 departments (89.3% vs 85.1%, p = 0.0014). Finally, participation rates differed significantly but only slightly between strategy and control schools: educational strategy (86.9% vs 84.3%, p = 0.003), screening strategy (84.2% vs 87.1%, p = 0.001) and for environmental strategy (84.6% vs 86.7%, p = 0.013).

As compared with participants, non-participants were significantly older (p = < 0.0001) and more often had unemployed parents (p = 0.0143) and school backwardness (p < 0.0001). Boys and girls participated equally. Adolescents' baseline characteristics, overall and by strategy, are presented in Table 3, 4 and 5 and were similar to the French grade 10 population [29]. Students were in the expected age range, with more than 70% aged 15 years old. In total, 36%, 70%, 53% and 50.1% of students had school backwardness, were in grade 10 in a general and technological school, were girls and the family head was employed, respectively, as compared with 20%, 67%, 54% and 41%, respectively, in the French grade 10 population [29].

**Table 3:** Baseline sociodemographic characteristics of students, overall and by assigned strategy

	**Overall^¶^**	**Educational** **strategy**		**Screening** **strategy**		**Environmental** **strategy**	
				
		**No**	**Yes**	**No**	**Yes**	**No**	**Yes**
	**N = 5,458**	**N = 2,483**	**N = 2,975**	**N = 2,771**	**N = 2,687**	**N = 2,794**	**N = 2,664**
	**%**	**%**	**%**	**%**	**%**	**%**	**%**
							
**Mean age (years)**	15.8*	15.7^¥^	15.7^¥^	15.7^¥^	15.7^¥^	15.7^¥^	15.7^¥^
**Gender (% girls)**	52.9	56.8	53.6	56.5	53.6	54.8	55.4
**General and technological course**	69.7	78.6	83.3	77.9	84.5	78.9	83.5
**School boarding status**							
Non-boarder	22.6	21.9	21.4	21.1	22.1	24.8	18.2
Half-boarder	68.2	70.4	68.4	68.5	70.1	66.1	72.7
Full Boarder	9.2	7.7	10.3	10.4	7.8	9.1	9.1
**Schooling**							
Classic	61.4	64.9	67.2	65.8	66.6	65.2	67.1
Advance placement at school	2.1	2.2	2.8	2.3	2.8	2.6	2.5
Late placement at school	36.4	32.9	29.9	31.9	30.7	32.2	30.4
**Residence (Rural)**	47.0	47.1	50.3	54.3	43.3	46.2	51.6
**Social and professional class of the family head**							
Farmers, shopkeepers, craftsmen, managers	7.3	7.8	8.3	8.6	7.6	7.2	9.0
Executives	12.6	14.4	13.7	11.1	17.0	15.8	12.1
Intermediate jobs	18.5	18.5	21.0	17.7	22.1	20.9	18.8
Employees, workers	50.1	50.4	46.2	52.3	43.9	46.4	49.9
Inactive (unemployed, retired)	11.4	8.9	10.8	10.4	9.5	9.7	10.2
Parents occupation**							
Neither of the 2 parents works	7.0	5.2	6.5	6.6	5.1	5.0	6.8
One of the 2 parents works	31.3	29.8	30.2	31.0	29.0	28.8	31.2
The 2 parents work	61.7	65.1	63.4	62.4	65.9	66.2	62.0
Family income level**							
Low	6.9	6.3	6.7	7.5	5.5	6.0	7.0
Average	33.3	33.6	34.4	33.9	34.2	34.4	33.7
High	59.8	60.1	58.9	58.6	60.3	59.6	59.3
Parental physical activity level**							
Low	54.9	53.1	53.1	54.3	51.8	52.7	53.4
Moderate	3.8	4.4	3.3	4.2	3.4	3.8	3.8
High	41.2	42.5	43.6	41.5	44.7	43.4	42.8
Parents considered overweight**	39.1	40.0	40.2	40.1	40.2	40.4	39.8

¶ Overall baseline characteristic parameters are estimated according to stratification and cluster design

¥ Standard Deviation = 0.7

* Standard Error of the Mean = 0.02

** Declared by adolescents

**Table 4:** Baseline nutritional attitudes and behaviours of students, overall and by the assigned strategy

	**Overall^¶^**		**Educational** **strategy**				**Screening** **strategy**				**Environmental** **strategy**			
					
			**No**		**Yes**		**No**		**Yes**		**No**		**Yes**	
	**N = 5,458**		**N = 2,483**		**N = 2,975**		**N = 2,771**		**N = 2,687**		**N = 2,794**		**N = 2,664**	
								
	**%/** mean*	SEM**	**%/** mean*	**SD^¥^**	**%/** mean*	**SD^¥^**	**%/** mean*	**SD^¥^**	**%/** mean*	**SD^¥^**	**%/** mean*	**SD^¥^**	**%/** mean*	**SD^¥^**
														
**Knowledge score (0-100)**	50.9	0.5	51.9	9.1	51.2	9.0	51.6	9.0	51.4	9.1	51.7	9.1	51.3	9.0
														
**Dietary guidelines followed**														
Fruits and vegetables (≥ 5^#^)	13.0		12.3		14.5		13.0		14.0		13.4		13.6	
Meats, eggs and fishes (1-2^#^)	94.7		94.7		95.1		95.0		94.8		95.2		94.6	
Sugary foods (2-3^#^)	33.3		35.2		33.1		32.6		35.5		33.4		34.7	
Dairy product (3-4^#^)	40.8		40.3		43.3		42.1		41.8		41.6		42.3	
Starchy foods (3 to 6^#^)	69.1		70.7		71.0		71.1		70.7		71.6		70.1	
Drinks (≥ 5^#^)	85.9		85.5		86,0		85.6		85.7		84.9		86.7	
Number of meals per week (21-28)	61.1		62.6		64.1		64.1		62.7		64.0		62.8	
														
**Nibbling**	70.9		70.0		68.2		69.3		68.8		67.5		70.7	
														
**Physical activities guidelines followed**	47.1		45.6		47.3		45.9		47.2		46.3		46.8	
														
**Number of nutritional guidelines followed**	4.3	0.04	4.3	1.3	4.4	1.3	4.3	1.3	4.3	1.3	4.4	1.3	4.3	1.3
≤ 2	9.3		8.1		8.2		7.9		8.5		8.1		8.3	
3	18.3		18.1		17.2		17.4		17.7		16.8		18.4	
4	30.0		30.6		27.9		30.1		28.2		30.1		28.1	
5	25.4		26.0		26.5		26.2		26.3		25.8		26.7	
6	12.7		12.6		15.1		14.1		13.8		14.4		13.5	
≥ 7	4.2		4.6		5.2		4.4		5.5		4.8		5.1	

¶ Overall baseline characteristic parameters are estimated according to stratification and cluster design

* Data are mean when SD is displayed or percentages

** Standard Error of the Mean

¥ Standard Deviation

# Number of daily servings recommended by nutritional guidelines

**Table 5:** Baseline health and anthropometric characteristics of students, overall and by the assigned strategy

			**Educational** **strategy**				**Screening** **strategy**				**Environmental** **strategy**			
					
	**Overall^¶^**		**No**		**Yes**		**No**		**Yes**		**No**		**Yes**	
	**N = 5,458**		**N = 2,483**		**N = 2,975**		**N = 2,771**		**N = 2,687**		**N = 2,794**		**N = 2,664**	
								
	**%/** mean*	SEM**	**%/** mean*	**SD^¥^**	**%/** mean*	**SD^¥^**	**%/** mean*	**SD^¥^**	**%/** mean*	**SD^¥^**	**%/** mean*	**SD^¥^**	**%/** mean*	**SD^¥^**
														
**Body mass index (kg/m^2^)**	21.7	0.1	21.5	3.3	21.7	3.6	21.7	3.7	21.5	3.3	21.6	3.6	21.6	3.4
														
**Body size (IOTF classification)**														
Thinness Grade 3	0.2		0.1		0.3		0.3		0.2		0.1		0.4	
Thinness Grade 2	0.8		0.5		0.7		0.6		0.7		0.7		0.6	
Thinness Grade 1	4.5		5.0		3.7		4.5		4.1		4.5		4.2	
Normal	74.9		76.1		76.0		74.7		77.4		75.6		76.5	
Overweight	14.9		14.6		14.9		15.7		13.9		15.0		14.6	
Obese	4.6		3.6		4.3		4.3		3.7		4.2		3.8	
														
**Waist circumference (cm)**	73.1	0.7	73.0	8.5	72.1	9.2	72.7	8.9	72.3	8.8	73.6	9.3	71.4	8.3
														
**High waist circumference (Canada classification)**	13.4		13.2		11.1		12.3		11.7		14.9		9.0	
														
**Eating behaviour disorders (EAT-40)**														
Low risk (< 17.5/100)	81.3		81.2		82.3		81.4		82.2		82.1		81.5	
Moderate risk (17.5/100 - 30/100)	9.9		9.8		10.1		9.9		9.9		9.2		10.7	
High risk (≥ 30/100)	8.8		9.0		7.6		8.7		7.9		8.7		7.8	
														
**Hospital Anxiety and Depression (HAD scale)**														
**High anxiety score (≥50/100)**	23.3		23.8		24.3		24.4		23.7		22.5		25.7	
**High depression score (≥50/100)**	3.2		2.8		2.5		2.8		2.6		2.6		2.7	
														
**Duke Health Profile**														
**Physical score (0-100)**	75.4	0.5	75.1	18.9	75.6	18.5	75.3	18.6	75.5	18.7	76.1	18.6	74.6	18.7
**Mental score (0-100)**	64.4	0.6	64.8	23.4	63.9	23.3	64.0	23.6	64.7	23.1	65.1	23.1	63.5	23.6
**Social score (0-100)**	68.8	0.4	68.7	19.2	68.6	19.3	68.5	19.4	68.8	19.1	68.9	19.1	68.4	19.4

¶ Overall baseline characteristic parameters are estimated according to stratification and cluster design

* Data are mean when SD is displayed or percentages

** Standard Error of the Mean

¥ Standard Deviation

Nutritional guidelines the most likely to be improved by interventions are those for fruits and vegetables (13.5%), limiting nibbling (29.1%), sugary foods (34.1%), dairy products (42.0%) and physical activity (46.6%), as well as the number of nutritional guidelines followed (< 27% did not follow at least two-thirds of the nutritional guidelines). The mean nutritional knowledge score was only about half the total score and suggests an opportunity for improvement, especially for the educational strategy.

Higher ICCs (> 0.100) were observed for age, kind of course (general and technological or vocational), type of schooling, residence (rural or urban), knowledge score and waist circumference (see additional file 1: ICC 1, for overall and by strategy). Stratification increased the power greatly for all outcomes except gender, kind of course, residence and waist circumference (see additional file 2: ICC 2, for overall and by strategy).

Most of the students were half-time boarders (n = 3,766, 68.2%) and more often lived in urban areas (n = 2,663, 47.0%); 50.1% had parents who worked and 59.8% declared a high family income.

At baseline, 14.9% of adolescents were overweight (n = 792) and 4.6% were obese (n = 215). The mean BMI was 21.1 kg/m^2 ^(standard error of mean (SEM = 0.1), and was higher in girls than in boys (respectively 21.8 kg/m^2 ^(SEM = 0.1) vs 21.6 (SEM = 0.1)) but the sexes did not differ in overweight and obese proportion. Concerning family nutritional environment, 54.9% of students declared a low parent physical activity, and 39.1% reported that their parents were overweight. Some students were at high risk of psychological troubles: 8.8% of students were at high risk of eating disorders, 23.3% anxiety and 3.2% depression.

## Discussion

The need for randomised trials of complex interventions such as health promotion are high, but such trials are a relatively new phenomenon [2,3], and their role is still not self-evident in public health nutrition research. Clustering, multiple factorial and long-term duration are particularly suited for health promotion trials intended to provide high-quality evidence to support public health policy [30]. Such trials allow for implementing interventions in real conditions within appropriately diverse populations from heterogeneous settings and reporting on a broad range of health outcomes.

Cluster randomised trials are a common and necessary design for assessing community interventions, especially when they involve environmental actions and rely on interactions between subjects. This type of trial has methodological difficulties [31,32] and is still not well reported [33]. We paid attention to the building of clusters for representativeness at the regional level and a minimal clustering effect through stratification; to the sample size calculation, taking into account several ICC estimates for each outcome [31]; and finally to the reporting process according to the CONSORT statement extended for cluster randomized trials [28]. A limitation of clustered randomised trials is that the interventions cannot be blinded. This potential bias is minimized since we ensured randomisation by high school, that only school nurses are responsible for anthropometric measures, and the factorial plan created a combination of interventions.

The PRALIMAP trial incorporates a rare 2 × 2 × 2 factorial cluster randomised design. The design was selected to evaluate all 3 strategies and their potential synergy. Factorial designs have been used in individual randomised trials, but combined with clustering, fewer than 10 were 2 × 2 designs, and to our knowledge, only 1 was a 2 × 2 × 2 design [34].

The PRALIMAP trial duration is in line with the Sharma et al. recommendations to provide interventions longer than 6 months [35,36]. The PRALIMAP interventions spread out over 24 months allows for drawing conclusions that are sustainable in the long run.

High schools were included in 2 waves, of 8 and 16 schools each. We chose this format because we were unable to implement the interventions and the measurements at the same time in the 24 schools spread over a 23,547-km^2 ^area and including more than 2,000 professionals to be informed and trained. This design appears to be superior to an experimental pilot site, through the dynamics created between the 2 waves of high schools and the ability to respect the randomisation plan (balance between strategies according to stratification criteria). A wave effect will be looked for and, if needed, taken into account in the analysis.

We chose adolescents as the target for the PRALIMAP intervention. During adolescence, individuals develop responsibility for health-related behaviours and attitudes that affect their future [37,38]. Moreover, eating habits initiated during this time are long-lasting [39]. Eventually, a strategy based on fostering personal responsibility, cognitive self-regulation and competence could be effective in improving healthful eating and physical activity behaviours among middle school children [40]. We did not involve parents in the intervention because adolescents' increasing independence around food choice is described as an act of parental defiance and peer solidarity [41]. Adolescents resolve the conflict between their need for autonomy over their food choices and the needs of others in the family by making their own meals, eating out, eating what is served, and negotiating to have their own and other family members' food choices and needs met [42]. This behaviour is one of the reasons why we chose a school-based intervention as opposed to family or community interventions. Another reason was that educational skills of professional teachers should increase the effectiveness of lessons introduced into the curriculum. The school has been described as an ideal place to run prevention interventions considered an integral part of the educational mission in France [43], as well as internationally [5,44], and to avoid known financial barriers in prevention access.

In terms of national and regional academic programs, schools are free to choose the form in which they provide education for their students, and nutritional education programs such as that in the PRALIMAP trial are used in many French schools without the need to obtain parental consent. Moreover, schools modify their environment on their own. Parental consent is required not for the intervention per se but for the outcomes measurements. Although schools were randomly assigned without consent from adolescents and their parents, both groups received information and could decline to participate in completing questionnaires and measuring body size. Information, access to data and the right to withdraw participation is warranted by French law after approval by adequate committees.

Summerbell et al. reported that studies focusing on the combination of dietary and physical activity did not show a significant improvement in BMI but that some studies focusing on dietary or physical activity alone showed a small but positive impact on BMI status; however, nearly all studies found some improvement in diet or physical activity behaviour [3]. In contrast, Sharma et al. showed that interventions targeting both physical activity and dietary behaviour were successful in influencing adiposity indices [36]. The PRALIMAP trial aims to focus on both diet and physical activity (as part of an integrative nutritional approach) in each of the 3 strategies.

Nutritional education has been evaluated in adolescents in a few studies with varying design and effectiveness [2,35,45]. A systematic review of published and "grey" literature [35] reported a moderate effect in adolescents 13-18 years of age. However, most of these interventions were classroom-based activities with an adapted curriculum. The PRALIMAP trial involves lectures in a quantity corresponding to the median yearly number of hours devoted to this task in other countries [6] but also experiments with other kinds of education through autonomous collaborative works and nutritional parties. These activities may lead to better motivation in students, thanks to a more convivial, pleasant and positive approach to nutrition and to skills acquisition and empowerment.

The PRALIMAP screening strategy is a new concept of school-based screening combining school-based measurements of BMI associated with waist circumference to define overweight and obesity and school-based care. At the onset of the trial, this new approach aroused debates among high school professionals who considered that the high school should not be viewed as a place for health care and among health professionals who are not well trained in proactive strategies of health problems management, being more comfortable with subjects contacting them directly with a care demand. However, Kubik et al. [46] described schools as a setting for obesity prevention (primary and secondary) and particularly highlighted the school nurses' responsibilities as vital but underutilized in delivering school-based obesity prevention. Moreover, the American Institute of Medicine recommended in 2005 to measure BMI in school and to report the results to parents [5]. Some school-based screening programs, practice, and effectiveness have been evaluated, and guidance has been provided for implementing such an approach [6,12,47].

In the PRALIMAP trial, waist circumference is associated with BMI to determine overweight and obesity because this measurement is convenient, simple to measure, and correlated with BMI, an approximate index of total body fat, and can be used for longitudinal assessment in management [44,48]. The measurement allows for avoiding false positives among athletic students in specific "sports and study" programs.

The proposed care of the PRALIMAP relies on the therapeutic education concepts in a stepwise collective approach that split up the intervention into several stages delivered by a multidisciplinary team, as recommended by the US Preventive Services Task Force [49]. Behavioural interventions were reported as probably safe in children 4-18 years of age and can be effective [50]. Barton et al. [49] showed that low-intensity interventions may be feasible for primary care but did not demonstrate a significant consistent benefit with regard to BMI. However, evidence is still insufficient because of the limited number and sample size of available studies. In the PRALIMAP trial, the intensity of the intervention is low (10-14 hours), but weight outcomes are long term and the number of subjects is high.

The environmental strategy implies reconsideration of high school nutrition policy and functioning, which can be difficult for school staff. However, since 2007, such strategies must be undertaken in French schools with regard to the new recommendations for catering [51]. Moreover, high schools implementing the environmental strategy were specially funded by the Regional Council to help them improve dietary and physical activity school facilities and offerings. Only a few studies have evaluated school nutritional environment interventions, but none showed conclusive results in terms of adolescents' body size, and one found a positive long-term effect on only dietary behaviour [2,35,35,45,52]. In the PRALIMAP environmental strategy, a new tool was used to help catering staff improve meals by use of special software that allowed for observing food consumption every day and better adapting the offerings for students, especially for fruits and vegetables [53].

The environmental strategy features an annual nutritional party, but its objectives differ from those of the education strategy party. In the environmental strategy, the party aims to help students discover new foods and physical activities to let them diversify their energy intake and expenditure, whereas the educational strategy party is a pedagogic way to evaluate and improve knowledge.

Multicomponent interventions promoting a healthy diet have been evaluated in high school-aged adolescents in European Union countries. These interventions were of limited effectiveness for self-reported dietary behaviour, and only one included anthropometric measurements, and results were inconclusive [35]. Eating patterns are more likely to improve when changes in the school environment are integrated with classroom nutrition education [54]. "Making healthy choices easier" is a strong recommendation for combining both strategies and needs to be evidenced [55]. In fact, no study has assessed the effects of environment and education strategies and their interaction. Because of its factorial design, PRALIMAP allows for determining an interaction between strategies and thus could provide information on an expected greater effectiveness of the combination of consistent educational and environmental strategies. Moreover, it allows for investigating a higher order interaction with the screening strategy. The combination of the 3 strategies could gather all conditions that could contribute to improving the prevention of overweight and obesity.

The PRALIMAP is a pragmatic trial where interventions were implemented in the real context of high schools. Effectiveness [56] is evaluated through body composition measurements, nutritional behaviour and knowledge outcomes, as was recommended by some authors [35,45,57]. The PRALIMAP outcomes rely on anthropometric data collected by high school nurses and on self-reported measures such as knowledge and behaviours collected in the classroom setting. At baseline, we achieved a high participation rate for both types of measurements (85.7%). The availability of the Board of Education database allows for comparison of the sociodemographic differences between participants and non-participants and suggests a probable higher non-participation rate among overweight students. However, non-participation in the PRALIMAP measurements is similar across arms. The turnover with entry level and attrition at each grade is common in schools and is likely to reduce power and even introduce bias if these data differ by randomisation arm. The design offers the opportunity to analyse the data in a longitudinal cohort approach, as well as in a repeated cross sectional approach. It offers the opportunity to combine hard outcomes (body size) with declared and perceived outcomes [58] (self-administered questionnaires) to explore the hypothetical cause from knowledge to health. Questionnaires used were standardised internationally or specifically designed for PRALIMAP. The time interval between the second and last visit measurements was shorter in the vocational than general and technological high schools, but the stratification design allowed us to control for this difference.

A remarkable feature of the PRALIMAP is the extensive and comprehensive process evaluation. The trial was designed to investigate not only the outcomes relating to nutritional behaviours but also the processes involved in developing and implementing the intervention strategies, as well as the type of nutritional activities provided in the control schools for each strategy. This feature can help interpret observed relationships between the interventions and outcomes. A specific work is planned to provide an estimation of the dose of intervention, which will allow for more intensive analysis beyond a simple interpretation task by performing a per protocol statistical analysis including the dose of each strategy implemented in each high school.

In conclusion, the PRALIMAP trial aims to improve the prevention and management of overweight and obesity in adolescents by translating current evidence into public health practice. For almost 10 years, the French government has implemented a concerted nationwide strategy to reduce the prevalence of obesity at every age. Determining the most effective strategies to implement guidelines in schools is a major component of this program, which needs to be more successful in meeting the needs of subjects, particularly those from lower socioeconomic classes [59]. The results should inform how best to implement effective nutrition prevention programs in a school environment targeting adolescents at a time in their lives when they develop responsibilities and empowerment for health attitude behaviours. The initial results are expected in late 2010.

## Competing interests

The authors declare that they have no competing interests.

## Authors' contributions

SB is the principal investigator for the PRALIMAP trial. JFC is the co-investigator. NA, EB, SB and ST are outcomes evaluation and statistical managers. EB, JFC and KL are the process evaluation managers. NA, EB, SB and ST drafted the manuscript. EL is logistic head managers and EA is high school professional head managers. All authors read and approved the final manuscript. SB is the paper guarantor. PRALIMAP trial group consisted of EA, EB, SB, EL and, LB, CG, AO and RDL. It has the power to make all strategic decisions and assures the cooperation between investigator teams and between field and investigator teams.

## Supplementary Material

Additional file 1 **ICC1: Intra-class correlation coefficient estimates without taking into account the stratification for high school administrative area department and type**. Click here for file

Additional file 2 **ICC2: Intra-class correlation coefficient estimates taking into account the stratification for high school administrative area department and type**. Click here for file
