# Supplementary material for: PRALIMAP: study protocol for a high school-based, factorial cluster randomised interventional trial of three overweight and obesity prevention strategies
Source: Trials. 2010 Dec 6;11:119. doi: 10.1186/1745-6215-11-119 (PMC3017023; doi:10.1186/1745-6215-11-119)
Supplement: Additional file 1 — ICC1: Intra-class correlation coefficient estimates without taking into account the stratification for high school administrative area department and type. [file 1745-6215-11-119-S1.PDF]

	Overall	Educational strategy No	Educational strategy Yes	Screening strategy No	Screening strategy Yes	Environmental strategy No	Environmental strategy Yes
Sociodemographic characteristics							
**Age**	0.195	0.208	0.193	0.202	0.203	0.202	0.202
**Gender**	0.091	0.084	0.103	0.071	0.119	0.140	0.047
**General and technological courses**	0.841	0.744	0.952	0.798	0.912	0.874	0.815
**Full boarder or half-boarder**	0.034	0.033	0.039	0.015	0.058	0.026	0.033
**Classic schooling**	0.117	0.117	0.126	0.101	0.146	0.150	0.092
**Residence (Rural)**	0.375	0.574	0.229	0.296	0.461	0.287	0.418
**Employee or worker family head**	0.056	0.065	0.054	0.044	0.066	0.066	0.051
**Both parents work**	0.039	0.038	0.043	0.027	0.054	0.050	0.028
**A moderate family financial level**	0.001	0.002	0.000	0.001	0.000	-0.001	0.003
**High parents physical activity level**	0.026	0.031	0.026	0.033	0.021	0.040	0.016
**Parents considered overweight**	0.005	0.006	0.004	0.005	0.005	0.000	0.010
Nutritonal attitudes and behaviours							
**Knowledge score (0-100)**	0.121	0.122	0.130	0.128	0.119	0.121	0.131
**Dietary guidelines followed**							
Fruits and vegetables (≥ 5[#])	0.015	0.018	0.013	0.023	0.010	0.012	0.021
Meats, eggs and fishes (1-2[#])	0.007	0.011	0.004	0.006	0.008	0.008	0.007
Sugary foods (2-3[#])	0.005	0.003	0.006	-0.001	0.009	0.006	0.004
Dairy product (3-4[#])	0.008	0.001	0.013	0.003	0.014	0.014	0.002
Starchy foods (3 to 6[#])	0.017	0.016	0.020	0.010	0.027	0.020	0.016
Drinks (≥ 5[#])	0.001	-0.003	0.004	0.004	-0.001	-0.001	0.002
Number of meals per week (21-28)	0.024	0.027	0.024	0.023	0.028	0.028	0.023
**Nibbling**	0.011	0.010	0.013	0.007	0.018	0.008	0.014
**Physical activity guidelines followed**	0.026	0.028	0.027	0.028	0.027	0.050	0.004
**Number of nutritional guidelines followed**	0.058	0.060	0.057	0.040	0.078	0.061	0.060
Health and anthropometric measurements							
**Body Mass Index (kg/m²)**	0.025	0.028	0.025	0.022	0.030	0.028	0.023
**Overweight and obesity**	0.004	0.008	0.001	0.002	0.005	0.004	0.005
**Waist circumference (cm)**	0.165	0.142	0.195	0.053	0.277	0.209	0.069
**High waist circumference**	0.060	0.071	0.054	0.017	0.114	0.081	0.012
**High risk of eating disorder (EAT-40)**	0.009	0.015	0.004	0.004	0.016	0.013	0.005
**High risk of anxiety (HAD scale)**	0.013	0.014	0.013	0.019	0.008	0.018	0.006
**High risk of depression (HAD scale)**	0.012	0.008	0.018	0.012	0.014	0.016	0.010
**Mental Duke score (0-100)**	0.025	0.027	0.025	0.035	0.016	0.042	0.008
**Physical Duke score (0-100)**	0.028	0.028	0.031	0.031	0.029	0.049	0.003
**Social Duke score (0-100)**	0.019	0.023	0.017	0.024	0.016	0.036	0.008
