# Supplementary material for: PRALIMAP: study protocol for a high school-based, factorial cluster randomised interventional trial of three overweight and obesity prevention strategies
Source: Trials. 2010 Dec 6;11:119. doi: 10.1186/1745-6215-11-119 (PMC3017023; doi:10.1186/1745-6215-11-119)
Supplement: Additional file 2 — ICC2: Intra-class correlation coefficient estimates taking into account the stratification for high school administrative area department and type. [file 1745-6215-11-119-S2.PDF]

	Overall	Educational strategy		Screening strategy		Environmental strategy	
		No	Yes	No	Yes	No	Yes
Sociodemographic characteristics							
**Age**	0.034	0.056	0.019	0.020	0.056	0.050	0.027
**Gender**	0.089	0.087	0.101	0.079	0.089	0.172	0.023
**General and technological courses**	0.218	0.055	0.157	0.112	0.499	0.249	0.253
**Full boarder or half-boarder**	0.036	0.026	0.017	0.017	0.040	0.039	0.037
**Classic schooling**	0.009	0.006	0.005	0.002	0.016	0.021	0.001
**Residence (Rural)**	0.345	0.396	0.269	0.293	0.456	0.196	0.376
**Employee or worker family responsive**	0.017	0.030	0.001	0.009	0.024	0.016	0.022
**Both parents work**	0.019	0.007	0.012	0.007	0.039	0.012	0.013
**A moderate family financial level**	0.000	0.001	-0.001	0.003	0.000	-0.001	0.001
**High parents physical activity level**	0.009	0.006	0.012	0.019	0.003	0.009	0.003
**Parents considered overweight**	0.003	0.006	0.001	0.006	0.000	0.001	0.006
Nutritonal attitudes and behaviours							
**Knowledge score (0-100)**	0.054	0.060	0.066	0.052	0.064	0.065	0.057
**Dietary guidelines followed**							
Fruits and vegetables (≥ 5[#])	0.015	0.018	0.019	0.031	0.011	0.012	0.030
Meats, eggs and fishes (1-2[#])	0.004	0.009	0.003	0.003	-0.004	0.008	0.002
Sugary foods (2-3[#])	0.002	0.002	0.004	-0.002	0.003	0.007	-0.001
Dairy product (3-4[#])	0.003	0.001	0.000	0.000	0.006	0.009	0.000
Starchy foods (3 to 6[#])	0.017	0.015	0.013	0.009	0.014	0.012	0.016
Drinks (≥ 5[#])	0.011	-0.001	0.007	0.007	-0.002	0.000	0.002
Number of meals per week (21-28)	0.002	0.018	0.009	0.013	0.007	0.018	0.007
**Nibbling**	0.011	0.003	0.006	0.005	0.004	-0.002	0.013
**Physical activity guidelines followed**	0.006	0.040	0.019	0.028	0.030	0.068	0.003
**Number of nutritional guidelines followed**	0.031	0.020	0.020	0.025	0.015	0.026	0.019
Health and anthropometric measurements							
**Body Mass Index (kg/m²)**	0.014	0.009	0.018	0.011	0.017	0.009	0.022
**Overweight and obesity**	0.002	0.008	0.004	-0.002	0.004	0.001	0.004
**Waist circumference (cm)**	0.106	0.095	0.140	0.034	0.130	0.093	0.058
**High waist circumference**	0.048	0.071	0.079	0.008	0.086	0.039	0.021
**High risk of eating disorder (EAT-40)**	0.009	0.015	0.004	0.005	0.018	0.016	0.006
**High risk of anxiety (HAD scale)**	0.014	0.014	0.023	0.025	0.013	0.020	0.006
**High risk of depression (HAD scale)**	0.003	0.008	0.021	0.005	0.005	0.006	0.003
**Mental Duke score (0-100)**	0.026	0.035	0.006	0.045	0.013	0.050	0.008
**Physical Duke score (0-100)**	0.029	0.034	0.018	0.038	0.032	0.052	0.005
**Social Duke score (0-100)**	0.018	0.029	0.014	0.028	0.015	0.036	0.009
